# Influence of Plant and Animal Proteins on Inflammation Markers among Adults with Chronic Kidney Disease: A Systematic Review and Meta-Analysis

**DOI:** 10.3390/nu13051660

**Published:** 2021-05-14

**Authors:** Danielle Francesca Aycart, Sofía Acevedo, Lucía Eguiguren-Jimenez, Jeanette Mary Andrade

**Affiliations:** Food Science and Human Nutrition Department, University of Florida, Gainesville, FL 32611, USA; danielle.aycart@ufl.edu (D.F.A.); sofiaacevedo@ufl.edu (S.A.); leguiguren@ufl.edu (L.E.-J.)

**Keywords:** chronic kidney disease, plant proteins, animal proteins, inflammation markers

## Abstract

Proteins, especially plant proteins, may reduce inflammation among adults with chronic kidney disease (CKD). This systematic review and meta-analysis were conducted to evaluate the effect protein types (animal or plant) have on inflammation markers (CRP, IL-6, TNF-α) among adults with varying stages of CKD. The Preferred Reporting Items for Systematic Review and Meta-analysis (PRISMA) was conducted to identify articles from inception until January 2021, utilizing six databases. Controlled trials that compared the effects of different protein types were analyzed using random-effects meta-analysis. Quality assessment and risk of bias of the included articles were assessed by using Cochrane risk of bias instrument and ROBINS-I. Out of the 10 studies that met the criteria, there was a decreasing trend in CRP levels when consuming plant proteins compared to animal proteins among non-dialysis participants. There was a statistically significant decrease when comparing animal proteins to unspecified proteins in CRP levels among dialysis participants [Hedges’ g = 2.11; 95% CI 1.12, 3.11; *p* ≤ 0.001], favoring unspecified proteins. Furthermore, animal proteins (eggs, red meat) showed increasing trends in CRP levels compared to whey protein isolate. Caution must be considered regarding these results as controlled, non-randomized, trials were included in the analysis, which may have contributed to high risk of bias. Future research should focus on protein types and the impact they have on kidney disease progression and inflammation markers.

## 1. Introduction

Chronic kidney disease (CKD) has long been considered a silent and neglected killer compared to more prominent non-communicable diseases such as cardiovascular disease and diabetes [1]. Globally, an estimated 697.5 million people or 9.1% of the population have been diagnosed with this disease [2]. Diabetes and high blood pressure are the most common underlying causes of CKD, although heart disease, obesity, family history, and age are also risk factors [2]. All these risk factors can alter the function and structure of the kidneys irreversibly over months or even years, resulting in death. 

CKD is characterized as a low-grade chronically inflamed state that has five progressive stages. In the first two stages, patients are asymptomatic, thus may go undiagnosed [2,3,4]. As the disease progresses, end-stage renal disease (ESRD) ensues in which one needs long-term dialysis or a kidney transplant. This chronic low-grade inflammation is attributed to the presence of cytokines, acidosis, and oxidative stress [5,6]. Detection of inflammation among adults with CKD is commonly identified by Tumor Necrosis Factor-alpha (TNFα), Interleukin-6 (IL-6) and C-reactive protein (CRP) (whose production is stimulated by IL-6) [2,4,7]. CRP, especially, has been associated with an increased risk of cardiovascular disease, cardiovascular morbidity, and mortality risk in this population [6,7,8,9,10,11,12]. Over half of adults with advanced stages (3–5) of CKD have elevated levels of CRP and the prevalence is even higher, at 35–65%, in adults undergoing chronic hemodialysis (CHD) [13,14], which leads to poor quality of life, osteoporosis, and depression, etc. [13]. Although the pathogenesis involved in the development of chronic inflammation in adults with CKD has not yet been fully elucidated, it has been described as a consequence of several factors which include increased production of proinflammatory cytokines, oxidative stress, chronic and recurrent infections, fluid overload, sodium overload, and gut dysbiosis [3,5,13]. Therefore, it is important to monitor the inflammation markers in adults with CKD to identify other comorbid processes such as infections, periodontal diseases, cardiovascular disease, and other conditions that may contribute to this inflammation [15,16,17]. There is not a single therapeutic strategy to address the chronic low-grade inflammation in adults with CKD, but several factors such as living a healthy lifestyle and consuming a balanced diet may decrease levels.

Medical nutrition therapy is an important part of CKD management and is typically combined with weight management and anabolic pharmacotherapy [2]. At later stages of CKD, individuals, who do not have diabetes, are instructed to decrease daily protein consumption to 0.28–0.43 g/kgBW with keto-analogues or 0.55–0.60 without keto-analogues [18,19]. As the disease progresses, and one is on dialysis, recommendations increase to 1.2 g/kgBW/day [19]. Protein recommendations focus on consuming high-quality proteins such as eggs and chicken to preserve kidney function and to minimize the risk of protein energy malnutrition [18,19]. Despite these guidelines, several meta-analyses show modest effects of low protein diets slowing the loss of kidney function, suggesting that the protein type may also play an important role [20,21,22,23,24,25].

Results from observational studies showed that consuming more than two servings daily of animal proteins (AP) such as red meat led to CKD or advanced the progression of this disease [26,27]. This may have been attributed to production of acids upon metabolizing red meat, which leads to increased production of uremic toxins, an elevation in inflammation markers (C-reactive protein (CRP), interleukin 6 (IL-6), *p*-cresyl sulfate, TNF-α), and oxidative stress [28,29,30,31]. On the other hand, studies have shown that adults who consumed more plant protein (PP) or plant-based diets such as Mediterranean, Dietary Approaches to Stopping Hypertension (DASH), or vegetarian, resulted in reduction of weight, glycated hemoglobin (HbA1C), low-density and high-density lipoproteins and total cholesterol and lower inflammation markers among adults with various chronic diseases [32,33]. Further studies have demonstrated that through the consumption of PP or plant-based foods there is a reduction in the incidence of CKD [34], inflammation markers (IL-6 and CRP) [35], uremic toxins, all-cause mortality, and hospitalization among the CKD population [20,36]. The potential mechanism of action is that, as the intake of plants is increased, and if possible as the sole source of energy and protein, the net dietary acid load is lowered. This, in turn, lowers inflammation and oxidative stress [37]. Even though recent studies suggest that PP may delay the progression of this disease [35,38,39], the Kidney Disease Outcomes Quality Initiative (KDOQI) does not include a recommendation for a particular protein type (plant vs. animal) due to limited powered randomized controlled trials [19]. Moreover, even though several meta-analyzes have shown the benefits of consuming a low-protein diet on the progression of CKD, few have focused on the protein types and the impact on inflammation markers among adults with CKD. Therefore, the purpose of this systematic review and meta-analysis was to evaluate the impact protein types have on inflammation markers among adults with varying stages of CKD.

## 2. Materials and Methods

### 2.1. Selection of the Studies

This systematic review and meta-analysis was conducted following the Preferred Reporting Items for Systematic Review and Meta-analysis (PRISMA) [40]. The study protocol was registered in the prospective register of systematic reviews database (registration code: CRD 42020220748). Neither humans nor animals were involved in this study, therefore no IRB approval was acquired. 

Potential relevant published peer-reviewed journal articles were initially identified based on nested keyword searches. This was then followed by reviewing the title and abstracts of the articles based on determined inclusion/exclusion criteria and finally an assessment of the relevant articles that were closely associated with the original intent of the search. Eligible articles were identified in an exhaustive electronic search through May 2020, utilizing the following databases: Pubmed/Medline, Embase, Cochrane Library, Scielo, Scopus, Clinical Trials.Gov (US National Library of Medicine, Bethesda, MD, USA).

One researcher (D.F.A.) used the following search MeSH terms, focusing on topics regarding CKD and protein intake: “Chronic kidney disease OR kidney disease OR kidney failure OR kidney insufficiency OR kidney function OR kidney dysfunction OR renal disease OR renal failure OR renal insufficiency OR renal function OR renal dysfunction [MeSH terms]” AND “Dietary protein intake or dietary intervention OR dietary animal protein OR dairy product OR eggs OR meat OR protein dietary supplement OR edible insects OR dietary plant protein OR vegan diet OR vegetarian diet OR high protein diet OR protein restriction diet [MeSH terms]” NOT “animal model OR rat OR mice OR rabbit OR cancer OR gut microbiome OR Mesoamerican Nephropathy OR Pregnant women OR children OR infant OR Urinary tract problems OR Polycystic Kidney Disease OR bacteria OR fungi [MeSH terms]”.

**Figure 1:**
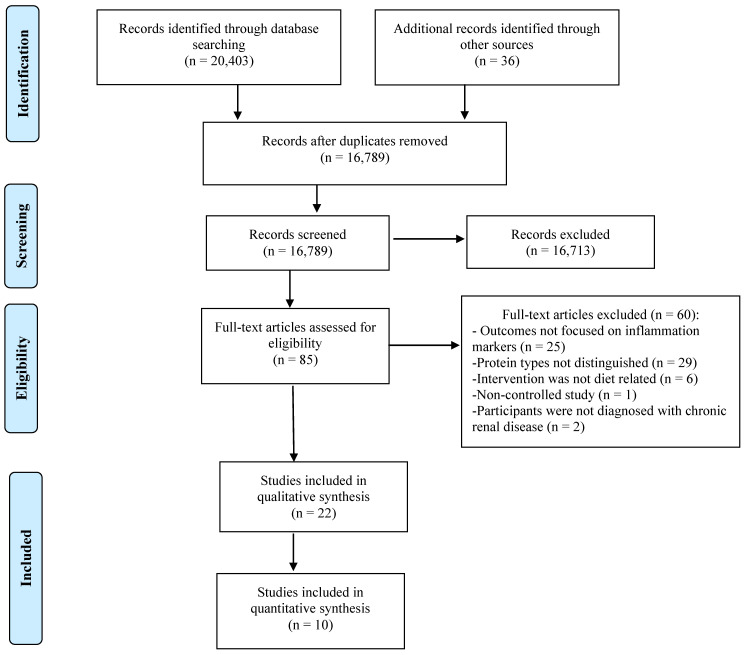
PRISMA flow diagram showing the screening process.

This combination of terms was carried out in the other databases using the search function for all topics. The researchers identified other relevant studies by searching the reference list of the retrieved studies, as well as conducting a second scan in January 2021. The search was completed for peer-reviewed articles written in English and Spanish with no time restrictions in order to encompass the vast literature available. Literature searchers were combined into DistillerSR (Evidence Partners, YOW, Canada), software to assist in screening and removing duplicate studies. Initially, 20,439 articles were identified for a total of 10 articles that could be included in this meta-analysis. Any discrepancies were discussed with the research team. See Figure 1 for a flow diagram of the article selection process.

### 2.2. Eligibility Criteria

Studies were included if they met the following criteria: (1) study design was controlled clinical trial (randomized control trial (RCT), controlled non-RCT, observational controlled); (2) included humans; (3) included participants over 18 years of age; (4) intervention included PP and/or AP; (5) included the amount and/or frequency that participants consumed these proteins; and (6) primary outcomes included inflammation markers such as CRP, IL-6, and/or TNF-α. Articles were excluded if the above criteria were not met. Additional, exclusion factors were non-peer reviewed articles, studies that were qualitative in design, conference abstracts, books, and unpublished registered clinical trials. 

### 2.3. Data Extraction 

All titles and abstracts were screened independently by two reviewers (D.F.A., S.A. and/or J.M.A.), and full-text studies that were considered relevant were included for further review. Three reviewers (D.F.A., S.A. and J.M.A.) independently reviewed all full-text articles, and any discrepancies were resolved with consensus. These discrepancies surrounded protein types and inflammation outcome variables. Data from the studies that fulfilled the eligibility criterion were collected onto Microsoft Excel: (1) first author’s last name and date of publication, (2) location and population size, (3) design and duration of the study, (4) intervention components, (5) inflammation marker measurement and (6) primary outcome(s). 

### 2.4. Quality and Risk of Bias Assessment

Quality and risk of bias assessment of the identified RCTs followed the Cochrane risk of bias instrument [41]. This tool consists of six sources of bias and assessed as high, low, or unclear based on the answer choices ‘yes,’ ‘no,’ or ‘unclear.’ The last category ‘unclear’ indicates either lack of information or uncertainty over the potential for bias. Based on the handbook, if the study was judged to be at low risk across all domains, it was overall deemed ‘low risk of bias.’ If the study was judged to have some concerns in at least one domain, it was determined as having ‘some concern.’ Finally, if the study was judged to be at high risk in at least one domain or judged to have some concern in multiple domains, it was deemed as overall ‘high risk of bias’ [41].

Quality and risk of bias assessment of the identified non-RCTs followed the Risk of Bias in Non-Randomized Studies–of Interventions (ROBINS-I) [42]. This instrument has seven domains of bias assessed as critical risk, serious risk, moderate risk or low risk, based on the answer choices of ‘yes’, ‘probably yes’, ‘probably no’, ‘no’, or ‘no information’ for each statement response. If a statement was answered with ‘yes’ or ‘probably yes’, subsequent statements were answered. Based on the statement responses, if no risk was apparent, the article was deemed as low risk of bias. Articles that were appropriate as a non-RCT but were not comparable to a RCT were considered low to moderate risk of bias. If an article was considered high risk for one domain, it was considered as at a serious risk of bias. If multiple domains were identified as high risk, then the article was deemed critical risk and needed to be removed from further analysis. For multiple domains identified as no information, the article would be considered at serious or critical risk and was removed from further analysis [42].

### 2.5. Assessment of Heterogeneity

A test of heterogeneity was performed when three or more studies were included in the meta-analysis. Assessment of statistically significant heterogeneity between primary outcome studies using the chi-square test and I^2^ statistic were completed. Statistical heterogeneity was determined at a chi-square (*p* < 0.01) and an I^2^ value of at least 50%. 

### 2.6. Data Synthesis

Studies were grouped based on intervention and mode of treatment (dialysis or not), AP group (intervention) and unspecified protein group (control) or PP group (intervention) and AP group (control) on inflammation markers. Following the guidelines provided by the Cochrane Handbook, non-RCTs were included due to the small number of RCTs available in the area of interest [41]. Data from each included trial were analyzed using Review Manager (RevMan, Version 5.4, Copenhagen, Denmark: The Nordic Cochrane Centre, The Cochrane Collaboration, 2020). Treatment effects were presented as the mean differences between changes and 95% confidence intervals (CI), and the pooled effects were computed by assigning each trial a weight of the reciprocal of its variance. If the raw data were unavailable, the variances for the changes of individual trials were calculated according to the methods described by the Cochrane Collaboration. A random-effect model was applied to determine differences. Though the outcomes evaluated in this meta-analysis have a limited number of trials, the funnel plots calculated using the Review Manager 5.4 were used to assess the presence or absence of publication bias for certain outcomes. The *p*-value threshold for statistical significance was set at 0.05 for effect size, and *p* ≤ 0.05 was considered significant.

## 3. Results

### 3.1. Study Characteristics 

Studies were conducted in Italy (*n* = 3) [43,44,45], United States (*n* = 3) [46,47,48], Brazil (*n* = 1) [49], China (*n* = 1) [50], Malaysia (*n* = 1) [51], and Portugal (*n* = 1) [52]. Study designs included RCTs (*n* = 4) [46,47,48,51], controlled cross-sectional (*n* = 1) [43], controlled, non-randomized trials (*n* = 4) [44,49,50,52], or a randomized crossover trial (*n* = 1) [45]. The total number of participants were 657, in which 77% were on CHD. The duration of the studies ranged from 1 to 18 months, with an average duration of 2 months as shown in Table 1.

All studies documented the amount of PP and/or AP within the intervention. Studies provided proteins based on kilogram of body weight (*n* = 3) [43,44,45] or based on gram weight of the product (*n* = 7) [46,47,48,49,50,51,52]. For the studies that offered a product, participants consumed it 90 minutes after initiation of hemodialysis treatment (*n* = 4) [49,50,51,52] or post-dialysis treatment (*n* = 3) [46,47,48]. Studies compared AP to unspecified proteins (*n* = 4) [49,50,51,52] or PP to AP (*n* = 6) [43,44,45,46,47,48]. Participants were encouraged to continue consuming their usual self-selected diet (*n* = 5) [44,46,48,49,52], provided a specific diet (*n* = 2) [43,45] or provided structured dietary counseling in addition to the consumption of the product (*n* = 3) [47,50,51]. Compliancy towards consuming the supplements or diet was determined via a variety of methods such as assessment through direct observation (*n* = 2) [46,49], returning empty or unused containers/packets (*n* = 3) [46,48,51], dietary records or recorded amount consumed of the supplement (*n* = 2) [45,51], 24-h urine (*n* = 2) [44,45] and/or blood samples [43]. Aside from one study [45], no study collected additional dietary information from the participants.

For the analysis of inflammation markers, studies were inconsistent in the methods to measure and analyze them. Studies either did not mention how blood samples were collected (*n* = 5) [44,45,47,51,52], or reported that they were collected pre- or post-dialysis treatments (*n* = 4) [46,48,49,50], or after an overnight fast (*n* = 1) [43]. Additionally, the methods of analysis for CRP levels were immuno-nephelometry (*n* = 3) [43,46,49], immunoturbidimetric assay (*n* = 1) [51], ELISA (*n* = 1) [48] or not specified (*n* = 5) [44,45,47,50,52]. For IL-6 and TNF-α, method of analysis was ELISA or enzyme immunoassay.

### 3.2. Quality and Risk of Bias

Based on the Cochrane risk of bias tool, four RCT studies [45,47,48,51] were considered high quality. Two studies [44,46] were considered unclear as regards selection bias as limited information was provided about the randomization process. 

Based on results from ROBINS-I, two studies were considered at low to moderate risk of bias due to comparability with RCTs, although information was lacking about how participants were randomized into the intervention and control groups [43,52]. Two studies were considered at moderate risk of bias due to the protocol for randomizing participants into the intervention and control group [49,50].

### 3.3. Publication Bias, Heterogeneity, and Risk of Bias

Visual inspection of the funnel plots revealed little evidence of asymmetry, and thus little evidence of publication bias (AP compared with non-specific protein CHD participants: CRP *p* < 0.001). 

For the studies that included CHD participants and compared AP with unspecified protein on CRP, the chi-square test indicated significant heterogeneity for between groups analysis (5.96; *p* < 0.000). For RCTs that included either non-CHD or CHD participants, chi-square and I^2^ were not reported for CRP, TNF-α and IL-6, as two studies or less focused on these inflammation markers with either PP and AP or AP and unspecified protein. Unclear risk domains predominated for selection bias due to inadequate details provided in the methodologies of the studies. 

### 3.4. Meta-Analysis 

#### The Effect of Protein Type on Inflammation Markers

The ten studies focused on inflammation markers based on protein types, measuring CRP (*n* = 10), TNF-α (*n* = 3), and IL-6 (*n* = 2). To minimize bias when combining RCTs with controlled, non-randomized trials in the meta-analysis, no analysis could be conducted with non-CHD participants as only two RCTs compared PP to AP [44,45], only two RCTs that included CHD participants used protein isolates as AP and PP [46,48], and only two RCTs compared AP to non-specified protein [47,51]. Results were measured between groups (*n* = 3) [49,50,52]. 

**Figure 2:**
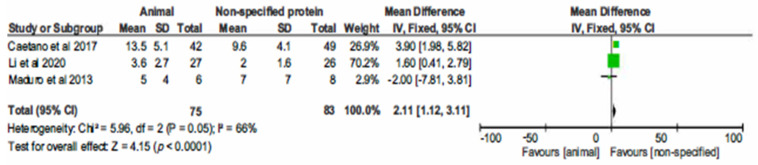
Forest plot of comparison: 1 Animal protein vs. unspecified protein, outcome: 1.1 CRP.

The three controlled, non-randomized, studies comparing AP to unspecified proteins in a total of 158 CHD participants demonstrated statistically significant difference in CRP levels, favouring the unspecified proteins (Hedges’ *g* = 2.11; 95% CI 1.12, 3.11; *p* < 0.001), as shown in Figure 2. 

Upon further analysis of each individual study, for the non-RCT [43] and two RCTs [44,45] that included non-CHD participants and compared PP to AP, there was a decreased trend in CRP (*n* = 1) [44] or statistically significant reductions in CRP levels (*n* = 2) [43,45]. In the RCT studies that included CHD participants and compared PP to AP, results showed no statistical difference in CRP, IL-6 and TNF-α levels [46,48]. For at least one of the studies, there was a decreased trend in CRP, IL-6 and TNF-α levels within and between groups, favouring the PP group [46]; whereas in the other study, there was an increased trend in CRP levels and a decreased trend in IL-6 levels among the PP group compared to the AP group [48].

For the RCT studies that included CHD participants and compared AP to non-specified protein, there was a non-statistical decrease in CRP levels favouring non-specified proteins [47,51]. Furthermore, there was a statistically significant increase in IL-6 levels, but a non-significant decrease in TNF-α levels in the non-specified protein group compared to the AP group [47].

## 4. Discussion

As has been stated before, plant proteins may have the ability to reduce inflammation markers among adults with CKD, more so than animal proteins. This systematic review and meta-analysis compared the effect of protein types on inflammation markers (CRP, IL-6, and TNF-α) among adults with varying stages of CKD. Results from the meta-analysis showed a statistically significant decrease in CRP levels when comparing AP to unspecified proteins, favouring the unspecified proteins. Further individual analysis of the studies showed that there was a decreased trend in CRP levels comparing the whey only AP snacks to the animal products that consisted of red meat [49], or a combination of eggs and milk proteins [50], or egg albumin, milk proteins and whey protein [52]. Moreover, a decreasing trend was observed in inflammation markers–CRP, IL-6 and TNF-α when comparing PP to AP [43,44,45,46,48].

Plant proteins contain between 5.7% (microalgae)–26.9% (wheat) of the amino acid glutamic acid and 80–100 mM of potassium within the cytoplasm of the plant [53]. When these PP are metabolized, they will consume hydrogen ions to minimize acid production [54]. Furthermore, more bicarbonate is produced during the breakdown of these PP, which reduces acid production [55]. This in effect leads to decreased production of uremic toxins, oxidative stress, and inflammation markers, which was found in two studies within this meta-analysis, from which only one was a RCT [43,45]. In Di the crossover randomized controlled study of Di Iorio et al (2018) among adults with CKD stages 3–4 (*n* = 60), participants were exposed to three different dietary regimens: a free diet for 3 months that consisted of 50–70 g of AP and 15–20 g of PP, a Mediterranean diet for 6 months that consisted of 30–40 g of AP and 40–50 g of PP, and a vegetarian diet for 6 months that consisted of 0 g of AP and 30–40 g of PP. Adherence was assessed through weekly dietary interviews and through 24-h weekly urine tests. Results showed that the vegetarian diet significantly reduced CRP levels compared to the free and Mediterranean diets. The researchers attributed this to the low serum levels of urea that suppressed the rise in inflammation markers [45].

On the other hand, Siefker et al. (2006) demonstrated an increasing trend in CRP levels when comparing a 4-day consumption of soy protein powder supplement (25 g/day) to whey protein powder (25 g/day) over 30 days among adults on CHD (*n* = 17) [48]. Soy protein, as opposed to other plant proteins, is considered a complete protein source and has less glutamic acid, at 12.4%, and more isoflavones [48] compared to other PPs [56]. As the researchers explained, for adults on CHD, the metabolism of soy is quite complex as the isoflavones remain in the blood and are unable to be cleared during dialysis, yet at the same time these isoflavones may reduce inflammation and oxidative stress [48,57]. Another reason for the results could be that participants were encouraged to consume high-quality protein foods during the intervention period. As the types of protein were not collected throughout the study period, participants may have been consuming AP in addition to the soy protein supplement [48], thus, the reason why there was a discrepancy in CRP levels when they were introduced to soy protein isolate as a supplement. Similarly, a recent meta-analysis of 12 studies with a total of 280 adults showed that there was no statistical difference in reducing CRP levels among adults on CHD, yet there was a decreasing trend in CRP levels among adults with later stages of CKD when consuming 14–30.7 g/day of soy or 35.5–61 mg/d of soy protein isolate [58]. However, as in the Siefker study, the meta-analysis did not mention if other sources of proteins were being consumed in addition to the soy [55]. Furthermore, as other studies have shown, consuming predominately PP daily may reduce the production of inflammation markers compared to consuming predominately AP daily [59,60,61].

In this systematic review and meta-analysis, an increasing trend was observed in CRP levels when participants consumed AP alone–red meat or in combination–egg albumin, milk proteins and whey protein. However, in one study that provided whey protein isolate as a supplement, results showed a decreasing trend in CRP levels [48]. In the 18-month randomized control trial of Sahathevan et al. (2018) a mong adults on CHD (*n* = 74), results demonstrated that consumption of 30 g of whey protein with nutrition counselling over 6 months showed a decreasing trend in CRP levels [51]. A recent meta-analysis of nine studies demonstrated that a decreasing trend in CRP levels was seen when adults with various chronic conditions (e.g., chronic obstructive pulmonary disorder) and diseases (e.g., cardiovascular and obesity) consumed >20 g of whey protein daily [62]. Whey protein is rich in amino acids-leucine (7.1%) and cysteine (0.8%). Leucine has anabolic effects that stimulate intramuscular protein synthesis through the upregulation of the mTOR pathway, which, in effect, reduces protein breakdown and induces anti-inflammatory effects [56,63,64,65]. Likewise, cysteine increases the synthesis of glutathione, which is considered an antioxidant that helps reduce inflammation [66]. This, though, was not observed in the 1-month randomized control trial of Siefker at al. (2006) among adults on CHD (*n* = 17). Results showed an increased trend in CRP levels when participants consumed whey protein supplement (25 g) compared to soy protein isolate (25 g) that was consumed four times a week for four weeks [48]. The discrepancies seen in the results of these studies may have been attributed to participants’ characteristics, dietary habits, and inflammation marker measurements.

Modest effects seen on inflammation markers may have been related to the participants’ characteristics. Participants within these studies were in the later stages of CKD pre-dialysis [43,44,45] or on CHD [46,47,48,49,50,51,52]. For the studies that included adults on CHD, an increasing trend in CRP levels was observed when comparing AP to unspecified proteins, or a decreasing trend in CRP levels when comparing PP to AP. An exception was noted in the study of Rhee et al. (2016) that showed a significant increase in IL-6 levels in the unspecified protein group (Group 2) compared to the AP group (Group 1) post-intervention (*p*= 0.002) [47]. While this was a novel finding, plausible explanations can be attributed to the types of proteins consumed. Furthermore, the CHD process itself contributes to low-grade chronic inflammation and, regardless of the type of protein consumed, will be catabolized [67]. This leads to protein-energy wasting (PEW), which increases mortality and morbidity risk [68,69]. As the focus of this meta-analysis was not on PEW or mortality/morbidity risk due to the proteins consumed, it was seen that during the CHD process, consuming AP daily (>20 g) as compared to PP contributed to a rise in CRP levels, although, the diet itself may also contribute to the elevation in inflammation markers, aside from just the protein types.

In the advanced stages of CKD, recommendations are to monitor potassium, phosphorus, sodium, and protein [37]. Foods rich in these nutrients, except for sodium, are fruits, vegetables, whole grains, beans and legumes, which aid in reducing production of inflammation markers and oxidative stress [51]. If adults with advanced stages of CKD do not consume these foods, this will contribute to elevation in inflammation markers. In this meta-analysis, only two studies focused on the types of food consumed [43,45]. Both studies showed a statistically significant decrease in inflammation markers when comparing results of participants with PP vs. AP. This demonstrates that the entire diet must be considered to have concise outcomes without the influence of other confounding dietary components. Moreover, with respect to dietary considerations in the different studies, only two included nutritional counseling. The results of these studies showed that there was not a statistical difference between intervention or control groups in CRP levels, which may have been related to compliancy toward consuming foods in order to improve kidney outcomes [50,70]. According to the literature, adults with CKD need medical treatment, which includes educating and counseling on diet. A cross-sectional study among adults with CKD (*n* = 399) associated perceived (think you know) to objective (actually know) nutrition knowledge with estimated Glomerular Filtration Rates (eGFR). Results showed that adults who had higher nutrition knowledge had a higher eGFR and less severe CKD compared to adults with lower nutritional knowledge [71]. Therefore, it is necessary for future studies to include nutritional counseling as a mechanism to reduce inflammation markers, along with monitoring dietary intake.

Another factor that contributed to the results were the methods used to collect and analyze the inflammation markers. In this meta-analysis, blood samples were used to analyze inflammation markers; however, studies were inconsistent regarding when and how these samples were collected. Four studies collected these labs’ pre-dialysis treatments, but did not specify if these samples were collected during fasting, or identify the amount and types of foods consumed prior to drawing these samples. The timing and frequency of consuming meals may have an impact on CRP levels. Based on a study of females (*n* = 2019) who were at high risk for breast-cancer risk, consuming 10% more calories after 5 pm the night prior to a morning blood draw resulted in elevated CRP levels [72], the reason why there may have been limited difference seen in inflammation markers between PP and AP proteins. Furthermore, a retrospective study examined the relationship between inflammation markers, CRP, TNF-α, and IL-6, and the risk of developing CKD after 15-years. At baseline, all the markers demonstrated a positive association with the prevalence of CKD, but at the 15-year follow-up, results showed only TNF-α and IL-6 were associated with the prevalence of CKD [73], which researchers attributed to other comorbidities such as diabetes and hypertension. However, Shankar et al. demonstrated that, when controlling chronic diseases such as hypertension and diabetes, they did not influence inflammation markers. Thus, the elevation in inflammation markers is likely to be due to CKD alone [72].

### Limitations and Strengths

To our knowledge, this is the first systematic review and meta-analysis that focused on identifying the impact protein types have on inflammation markers among adults with varying stages of CKD. This systematic review and meta-analysis do have limitations. The inclusion criteria targeted a specific chronic disease and specific outcomes, thus limited studies were included. Additionally, the inclusion of controlled, non-randomized, trials in the meta-analysis, may have contributed to high risk of bias. Furthermore, these trials were heterogeneous regarding their study design, number of participants, evaluation measures and techniques used, thus reducing ability to compare. One of the strengths of this review is that there was evidence to suggest that AP contributed to elevations in inflammation markers and that PP may reduce these. This pinpoints research priority areas and specific methodologies to improve the consistency of clinical data in future research. This meta-analysis showed that protein types and amounts need to be considered when examining the progression of CKD, especially on inflammation markers.

## 5. Conclusions

In conclusion, results from this meta-analysis showed that animal proteins compared to unspecified protein types increase CRP levels, and that there was a decreasing trend in inflammation markers when comparing plant proteins to animal proteins among adults with varying stages of CKD. However, the literature does not confidently portray this due to various confounding factors. Future research should focus on RCTs to compare protein types and their subsequent effect on inflammation markers among the CKD population to aid in guiding researchers and practitioners.

## Figures and Tables

**Table 1:** Data extraction from the included studies (*n* = 10) for the meta-analysis.

Author (Year)	Location and Sample Population.	Study Design and Duration	Study Intervention.	Inflammation Markers * and Collection Timing.	Methods/Techniques Used for Inflammation Markers.	Primary Outcome.
Bergesio et al. (2005) [43]	Florence, Italy. Adults on stable chronic kidney disease with moderate to severe renal failure (*n* = 60): Control group (*n* = 31). Intervention group (*n* = 29).	Controlled cross sectional. 3 up to 12 months.	Control group: 0.6 g/kg/day of protein (animal and plant protein). Intervention group: 0.3 g/kg/day of plant-based protein and one tablet per 5–8 kg/day of essential amino acids.	C-reactive protein (CRP). Collected after overnight fast.	Immuno-nephelometry.	Significant decrease between control and intervention group post- intervention (*p* < 0.05).
Siefker et al. (2006) [48]	Ohio, United States. Adults on Hemodialysis (*n* = 17): Control group (*n* = 9). Intervention group (*n* = 8).	Double-blind, randomized control trial. 1 month.	Control group: whey protein powder (25 g protein). Intervention group: soy protein powder (25 g protein).	CRP TNF-α. Collected before dialysis treatment.	CRP: ELISA TNF-α: enzyme immuno-assay.	CRP: No significant differences within groups (*p* > 0.05). Non-statistical increase within both intervention and control group post-intervention. TNF-α: No significant differences within groups (*p* > 0.05). Non-statistical decrease within the intervention group post-intervention.
Fanti et al. (2006) [46]	Texas, United States. Adults on Hemodialysis (*n* = 32): Control group (*n* = 13). Intervention group (*n* = 19).	Double-blind, randomized controlled trial. 2 months.	Control group: isoflavone-free milk-based supplements. Intervention group: isoflavone containing soy-based nutritional supplements.	CRP IL-6 TNF-α. Collected before dialysis treatment.	CRP: Immuno-nephelometry IL-6, TNF-α: ELISA	No significant difference, based on medians, within intervention and control group for CRP, IL-6 and TNF-α. Non-statistical decrease between pre- and post-intervention group for CRP, IL-6, and TNF-α.
Maduro et al. (2013) [49]	Sao Paulo, Brazil.Adults on Hemodialysis (*n* = 14): Group 1 (*n* = 8). Group 2 (*n* = 6).	Controlled, open-label prospective trial.1 month.	Group 1: Pre-intervention: meatless protein snack (2 g protein) Intervention: animal-based snack (29 g protein). Group 2: Pre and during intervention: animal-based snack (29 g protein).	CRPCollected before and after dialysis treatment.	Immuno-nephelometry.	Non-significant increase between groups 1 and 2 post-intervention.
Rhee et al. (2017) [47]	California, United States. Adults on Hemodialysis (*n* = 110): Group 1 (*n* = 55). Group 2 (*n* = 51).	Double-blind, randomized controlled trial. 2 months.	Group 1: 50–55 g animal-based protein, 850 kcal, 400–450 mg of natural phosphorus. Group 2: <1 g plant-based protein, <50 calories, <20 mg phosphorus.	CRP IL-6 TNF-α Collection time unknown.	CRP: ** IL-6, TNF-α: enzyme immunoassay	CRP: Non-significant decrease between group 2 and 1 post- intervention (*p* = 0.74). IL-6: significant increase between group 2 and 1 post-intervention (*p* = 0.002). TNF-α: Non-significant decrease between group 2 and 1 post-intervention (*p* = 0.35).
Caetano et al. (2017) [52]	Lisbon, Portugal. Adults on Hemodialysis (*n* = 91): Control group (*n* = 50). Intervention group (*n* = 41).	Non-randomized controlled study 6 months.	Control group: snack brought from home. Intervention group: 160 mL of a drink rich in high biological value protein (65% pasteurized egg albumin, milk proteins and whey proteins) and an egg sandwich.	CRP Collection time unknown.	**	No significant difference within intervention group (*p*= 0.48) or control group (*p* = 0.74). In the intervention group, non-statistical increase post-intervention (+2.4 mean).
Di Iorio et al. (2017) [44]	Avellino, Italy. Adults on stages 3 to 4 of Chronic kidney disease (*n* = 146): Control group (*n* = 92). Intervention group (*n* = 54).	Randomized, open label, controlled study. 12 months.	Control group: animal-based proteins, 0.6–1 g protein/kg/day. Intervention group: plant-based proteins, 0.3–0.4 g protein/kg/day, amino acid supplementation	CRP Collection time unknown.	**	Non-statistical decrease post-intervention between intervention group compared to control group (*p* > 0.05).
Di Iorio et al. (2018) [45]	Avellino, Italy. Adults on stages 3 to 4 of Chronic kidney disease (*n* = 60): Group A (*n* = 30). Group B (*n* = 30).	Prospective, randomized, crossover-controlled trial. 18 months	Free Diet (FD): proteins 1 g/kg body weight (bw)/day (animal proteins 50–70 g/day, plant-based proteins 15–20 g/day). Mediterranean diet (MD): proteins 0.7–0.8 g/kg (bw)/day (animal proteins 30–40 g/day, plant-based proteins 40–50 g/day). Very-low protein diet (VLPD): proteins 0.3–0.5 g/kg (bw)/day (animal proteins 0 g/day, plant-based proteins 30–40 g/day). Group A: 3 months FD/6 months VLPD and 3 months FD/6 months MD Group B: 3 months FD/6 months MD and 3 months FD/6 months VLPD.	CRP Collection time unknown	*	Significant decrease post- intervention between FD and VLPD (*p* = 0.01).
Sahathevan et al. (2018) [51]	Kuala Lumpur, Malaysia. Adults on Hemodialysis (*n* = 74): Control group (*n* = 37). Intervention group (*n* = 37).	Multicenter, parallel, open label randomized controlled trial. 18 months.	Control group: nutrition counseling only. Intervention group: whey protein supplement (15 g protein) and nutrition counseling.	CRP Collection time unknown.	Immuno-turbidometric assay.	Non-significant decrease within or between intervention and control groups post-intervention (*p* = 0.87).
Li et al. (2020) [50]	Baotou, China. Adults on Hemodialysis (*n* = 53): ∙ Control group (*n* = 27). ∙ Intervention group (*n* = 26).	Controlled, open-label, prospective trial. 3 months intervention + 3 months follow up.	Control group: nutritional counseling only, liberalized diet. Intervention group: nutritional counseling plus three intradialytic, protein-rich, animal-based meals 90 min after initiation of each HD session.	CRP Collected before dialysis treatment.	**	Non-statistical increase between groups after 6 months (*p* = 0.36).

* The inflammation markers measured in the studies were C reactive protein (CRP), Interleukin-6 (IL-6), and Tumor necrosis factor alpha (TNFα). ** Does not specify method used to determine inflammation marker.

## Data Availability

No new data were created or analyzed in this study. Data sharing is not applicable to this article.
